# Lane-Hamilton Syndrome in an Adult With Down Syndrome: A Case Report

**DOI:** 10.7759/cureus.33385

**Published:** 2023-01-05

**Authors:** Joana Fontes, Bárbara Sousa, Marta Moreira, Nuno Pardal, Rafael Lopes Freitas

**Affiliations:** 1 Internal Medicine, Unidade Local de Saúde do Alto Minho - Hospital Conde de Bertiandos, Ponte de Lima, PRT; 2 Gastroenterology, Unidade Local de Saúde do Alto Minho, Viana do Castelo, PRT; 3 Internal Medicine, Unidade Local de Saúde do Alto Minho, Viana do Castelo, PRT

**Keywords:** lane-hamilton syndrome, idiopathic pulmonary hemosiderosis, celiac disease, down syndrome, young adult male

## Abstract

The Lane-Hamilton syndrome (LHS) is an extremely rare association between celiac disease (CD) and idiopathic pulmonary hemosiderosis (IPH), with only a few cases reported in the literature.

The authors report a case of a 32-year-old man with Down syndrome who presented to the emergency department with a history of hemoptysis and chronic diarrhea. The complementary investigation revealed iron deficiency anemia and features suggestive of diffuse alveolar hemorrhage on computed tomography (CT) scan of the chest. After excluding all competing diagnosis, the IPH diagnosis was made. The biopsy of the small intestine confirmed CD and the diagnosis of LHS was established. A gluten-free diet and steroids were given to the patient with a very good clinical response.

Since there is an established association between IPH and CD, if the diagnosis of IPH is made, it's recommended to screen for CD even in patients without gastrointestinal symptoms.

## Introduction

Celiac disease (CD) is an immune-mediated inflammatory disease of the small intestine triggered by gluten ingestion in genetically predisposed individuals [1]. The clinical spectrum is highly variable ranging from gastrointestinal symptoms to extraintestinal manifestations, such as cutaneous (dermatitis herpetiformis), hepatic (celiac hepatits), neurologic (gluten ataxia), musculoskeletal (arthritis and myopathy) manifestations, and other rare ones like thromboembolic events [2].

The prevalence of CD has increased over the last five decades, partly due to better diagnostic tools and better screening of high-risk patients. The worldwide prevalence of CD is approximately 1.4% based on serologic tests and 0.7% based on histologic results, with higher incidence in females and children [3,4].

First- and second-degree relatives of patients with CD are at increased risk [5] as well as individuals with other diseases such as autoimmune disorders (eg. type 1 diabetes mellitus, autoimmune thyroiditis), Down and Turner syndromes [6] and idiopathic pulmonary hemosiderosis (IPH) [7].

The etiology of IPH remains unknown and, typically, manifests as a triad of recurrent hemoptysis, diffuse parenchymal infiltrates on chest radiography and iron deficiency anemia [8]. The association between IPH and CD was first described in 1971 by Lane and Hamilton. This combination, known as Lane-Hamilton syndrome (LHS), is extremely rare and, to date, no more than 80 cases have been reported [7].

The authors report a case of a 32-year-old man with Down syndrome presenting with hemoptysis and history of malabsorption in whom the diagnosis of CD and IPH was made.

## Case presentation

A 32-year-old non-smoker man with Down syndrome and a medical history of epilepsy presented to the emergency department due to a three-day history of hemoptysis. He denied fever, sputum production and chest pain. Three months prior to admission he started to experience diarrhea associated with marked abdominal bloating and weight loss. He had a medical history of diarrhea five years before. At that time, the initial investigation was normal (including full blood count, celiac serology [tissue transglutaminase and endomysium tests], thyroid function tests, human immunodeficiency virus [HIV] test and stool cultures) and, since the diarrhea was self-limited, it was decided not to proceed with additional investigation.

At admission the patient was alert, afebrile, hemodynamically stable with blood pressure 102/63mmHg and heart rate 65 beats per minute, and with no signs of respiratory distress with oxygen saturation 94% on room air. On examination, the lungs were clear on auscultation and the heart rhythm was regular without murmurs. There was evidence of malnutrition, weighing 31 Kg (body mass index: 14 kg/m2), marked pallor and abdominal distention.

**Figure 1:**
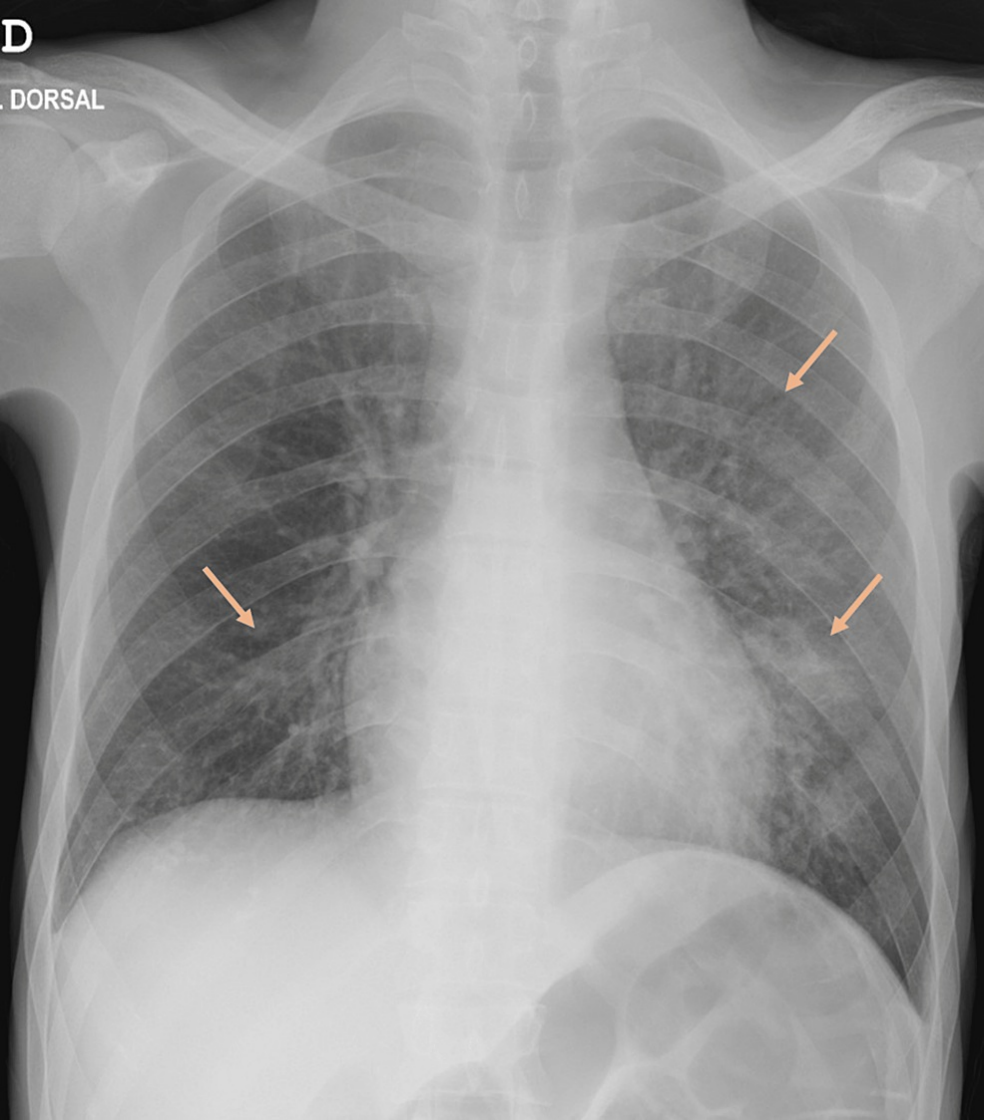
Chest X-ray showing bilateral interstitial infiltrates (arrows).

**Figure 2:**
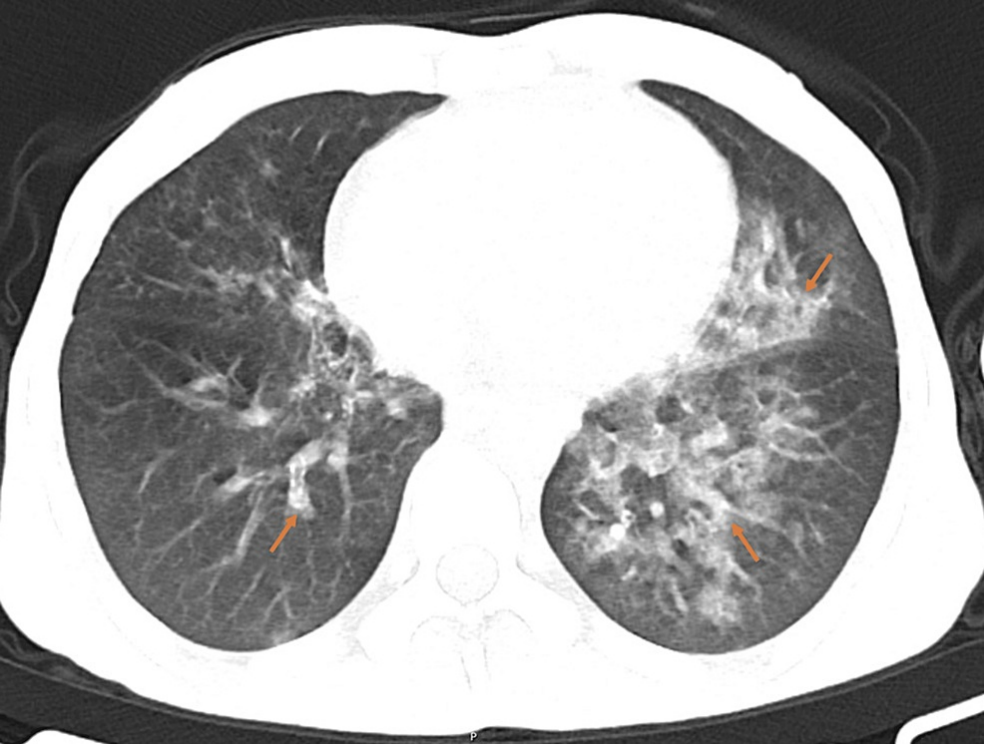
Axial computed tomography (CT) scan of the thorax showing bilateral patchy ground-glass opacities (arrows).

His arterial blood gas was normal and the initial laboratory results showed normocytic normochromic anemia (hemoglobin 11.3g/dL, hematocrit 31.9%, mean corpuscular volume [MCV] 86fL), hypokalemia (K 3.3mmol/L), C-reactive protein level of 6.67mg/dL and coagulopathy (international normalized ratio [INR] 2.02). The chest radiograph showed bilateral pulmonary interstitial infiltrates (Figure 1). A computed tomography (CT) of the thorax was performed and showed bilateral patchy ground-glass opacities suggestive of alveolar hemorrhage (Figure 2).

He was hospitalized in an internal medicine unit for symptom control and further investigation.

The laboratory investigation (Table 1) revealed low serum iron, hypophosphatemia, hypoalbuminemia and high erythrocyte sedimentation rate (ESR). The peripheral smear blood showed mild anisocytosis, anisochromia and hypochromia. The urinalysis was negative for hemoglobin, protein or red blood cells casts and the stool samples collected for Clostridium difficile analysis were also negative. The serologic tests were positive only for deamidated gliadin peptide-immunoglobulin A (DGP-IgA).

**Table 1:** Complementary laboratory results. ESR - erythrocyte sedimentation rate; TSH - thyroid stimulating hormone; HIV - human immunodeficiency virus; IgA - immunoglobulin A; IgG - immunoglobulin G; IgM – Immunoglobulin M; ANA - antinuclear antibodies; ANCA - antineutrophil cytoplasmic antibodies; Anti-dsDNA - antidouble stranded DNA antibodies; Anti-GBM - antiglomerular basement membrane antibodies; tTG - tissue transglutaminase; EMA - endomysial antibody; DGP - deamidated gliadin peptide

Laboratory test	Results (unit)	Reference range
Phosphorus	1.90 mg/dL	2.5-4-9
Magnesium	1.83 mg/dL	1.6-2.6
Corrected Calcium	8.0 mg/dL	8.6-10.3
Albumin	2.7 g/dL	3.5-5.0
Total protein	5.8 g/dL	6.4-8.2
ESR	113 mm/h	2-8
Iron	38 ug/dL	70-180
Ferritin	112.3 ng/mL	21.81-274.66
Acid folic	3.4 ng/mL	3.1-205
Vitamin B12	331 pg/mL	187-883
25-Hydroxycalciferol	19.1ng/mL	30-100
TSH	2.66 uUI/mL	0.35-4.94
HIV	non-reactive	-
IgA	808 mg/dL	60-400
IgG	994 mg/dL	700-1600
IgM	38 mg/dL	40-230
ANA	negative 1/160	-
ANCA	negative 1/20	-
Anti-dsDNA	negative 1/10	-
Anti-GBM	negative	-
Rheumatoid factor	<3.5 IU/mL	<30
tTG IgA	6.9U/mL	positive > 10
tTG IgG	1.8U/mL	positive > 10
EMA	negative 1/5	-
DGP IgA	43U/mL	positive > 10
Lupus anticoagulant	1.1	0.8-1-2
Anti-cardiolipin antibody IgG/IgM	negative	-
Anti-beta2 glicoprotein I IgG/IgM	2.6 / <0.90U/mL	>10 / >10

An ECG and echocardiogram were performed and reported as normal, excluding pulmonary hypertension or valvular disease.

A flexible bronchoscopy and bronchoalveolar lavage (BAL) were done. The macroscopic findings were normal; the microbiological culture of BAL didn’t show evidence of bacterial, fungus or mycobacterium growth; and BAL fluid cytology showed numerous alveolar macrophages and was negative for malignancy. No lung biopsy was performed.

Simultaneously, due to high clinical suspicion of celiac disease, the patient was started on a gluten-free diet (GFD) and underwent endoscopic study. The ileocolonoscopy was normal and the upper endoscopy revealed marked villous atrophy and nodular appearance in the first and second parts of duodenum. The histologic report of duodenal biopsy showed subtotal villous atrophy, crypt hyperplasia, and increased intraepithelial lymphocytes consistent with celiac sprue (Marsh III b [9]).

So, after excluding secondary causes of diffuse alveolar hemorrhage and confirming celiac disease, the diagnosis of idiopathic pulmonary hemosiderosis was presumed, corresponding to the Lane-Hamilton syndrome. 

The symptoms of the patient didn’t improve with GFD. As he was malnourished and didn’t tolerate oral diet, total parenteral nutrition and steroids were given. The patient responded very well to the initiation of corticosteroid therapy. The gastrointestinal symptoms improved, he was able to gain weight and the episodes of hemoptysis didn’t recur during five months of follow-up.

## Discussion

CD is a common cause of malabsorption worldwide that can occur at any age. Only a minority of patients have classical symptoms related to malabsorption (diarrhea, loss of appetite, abdominal distention), being more common in children. A much larger number of individuals have extraintestinal manifestations which mainly include iron deficiency anemia, osteopenia, hypertransaminasemia, infertility and a wide array of neurological symptoms. Rarely, it may present with thromboembolic events or it can even be silent as well [2,10]. The diagnosis is based on clinical data, serological tests and histological evaluation of small-bowel biopsies [10]. 

According to the literature, CD is more prevalent in Down syndrome patients compared to the general population, with an estimated prevalence of at least 5.4% [11]. Nevertheless, gastrointestinal disorders, anemia and failure to thrive are also very common in Down syndrome patients without CD which may predispose to a delayed or missed diagnosis [12]. In this clinical case, although the patient already had a history of diarrhea, the diagnosis of CD was made only with the recurrence of gastrointestinal symptoms in a more severe form. This portrays the importance of exhaustively screening these high-risk patients.

The prevalence of IPH among CD patients is not known and mostly affects the pediatric population [8]. Down syndrome patients also appear to have a higher risk of developing IPH [13]. IPH seems to be immune-mediated but the physiopathology remains to be established [8].

The diagnosis of IPH requires the exclusion of other disorders in which diffuse alveolar hemorrhage is a cardinal sign, such as autoimmune diseases, cardiac and coagulation disorders, infections or drug reactions. In addition to clinical presentation, the diagnosis is supported by compatible laboratory results, chest radiography and histopathologic findings. Laboratory work-up may show iron deficiency anemia, normal to elevated ferritin due to pulmonary iron overload and negative serologic tests for autoimmune disease and vasculitis. Chest radiography generally shows diffuse alveolar opacities in the acute phase. Sputum sample or BAL may show hemosiderin-laden alveolar macrophages on Prussian blue stain. Lung biopsy is the gold standard for the diagnosis and the histopathology usually reveals blood-filled alveolar spaces, free and intracytoplasmic hemosiderin macrophages, alveolar thickening with hemosiderin deposition, type 2 pneumocyte hyperplasia and absence of capillaritis and immune complex deposition [8].

Since there is an established association between IPH and CD, it is recommended to screen for CD even in patients without gastrointestinal symptoms [14].

In the reported case, the history of chronic diarrhea and hemoptysis led to the clinical suspicion of Lane-Hamilton syndrome. Unfortunately, the lung biopsy wasn’t performed but the clinical presentation and the complementary exams supported the diagnosis. The patient was discharged from the hospital with GFD and steroids therapy due to refractory disease.

Tryfon et al. conducted a rigorous search of international databases from 1971 to 2020 and found 80 cases with LHS diagnosis - 44 children and 36 adults. The most common symptoms reported were hemoptysis, dyspnea and cough, while anemia and iron deficiency were the most predominant biochemical findings. As described in this case, diffuse ground-glass opacities were the preeminent CT findings in adults. Treatment differed among cases, which makes it difficult to analyse the clinical response to each therapy. In the adult population, during the acute phase of the disease, 63.9% of patients were initiated on GFD and steroids were administred in 50% of patients. After discharge from the hospital, 86.1% of adult patients were indicated to GFD and in nine patients (25%) chronic steroids therapy with gradual tapering was implemented. The follow-up period was noted only in 16 cases, whereas seven cases required further treatment with steroids or azathioprine due to relapse or refratory disease. Of note, two patients with poor compliance with GFD relapsed even under azathioprine or high dosage of prolonged steroid therapy, which supports the importance of GFD [7].

To date, the mainstay for the treatment of LHS is a GFD, along with or without steroids or immunosuppressive therapy. It’s difficult to assess the prognosis due to the small series of patients and inadequate follow-up. The early recognition of this entity is important because a gluten-free diet has a positive effect on the regression of both CD and IPH [14].

## Conclusions

Idiopathic pulmonary hemosiderosis is a rare clinical entity of unknown etiology, even rarer in adults. Coexistence of celiac disease has been reported (called Lane-Hamilton syndrome), so all patients with the diagnosis of idiopathic pulmonary hemosiderosis should be screened for celiac disease even in the absence of gastrointestinal symptoms.

The diagnosis of Lane-Hamilton syndrome is important because a gluten-free diet, along with steroid therapy, seems to improve both pulmonary and gastrointestinal manifestations.
